# Hierarchical Structure and Catalytic Activity of Flower-Like CeO_2_ Spheres Prepared Via a Hydrothermal Method

**DOI:** 10.3390/nano8100773

**Published:** 2018-09-29

**Authors:** Genli Shen, Mi Liu, Zhen Wang, Qi Wang

**Affiliations:** CAS Key Laboratory of Standardization and Measurement for Nanotechnology, National Center for Nanoscience and Technology, Beijing 100190, China; shengl@nanoctr.cn (G.S.); liumi@nanoctr.cn (M.L.)

**Keywords:** ceria, catalytic activity, hierarchical structure

## Abstract

Hierarchical CeO_2_ particles were synthesized by a hydrothermal method based on the reaction between CeCl_3_·7H_2_O and PVP at 270 °C. The flower-like CeO_2_ with an average diameter of about 1 μm is composed of compact nanosheets with thicknesses of about 15 nm and have a surface area of 36.8 m^2^/g, a large pore volume of 0.109 cm^3^/g, and a narrow pore size distribution (14.9 nm in diameter). The possible formation mechanism of the hierarchical CeO_2_ nanoparticles has been illustrated. The 3D hierarchical structured CeO_2_ exhibited a higher catalytic activity toward CO oxidation compared with commercial CeO_2_.

## 1. Introduction

CeO_2_ is playing important roles in various fields such as promoters for three-way catalysts [1], fuel cells [2], hydrogen storage materials [3], and oxygen sensors [4]. Although the utilization of ceria is based on its intrinsic chemical properties, the structures and morphologies of CeO_2_ also have a significant influence on its properties and applications [5,6].

So far, CeO_2_ with different sizes and morphologies such as nanorods [7], nanospheres [8], nanotubes [9], and nanocubes [10] have been synthesized in the last decade. It was proved that CeO_2_ nanoparticles with different sizes and morphologies have better properties than general CeO_2_ does. CeO_2_ nanoparticles afford more active sites because of their high specific surface areas and novel structures [11].

Preparation of CeO_2_ with different structures and morphologies provides the basic groundwork for its advanced applications. Hierarchical structured CeO_2_ with unique properties and novel functionalities has attracted the attention of many researchers in recent years.

Zhong et al. synthesized a three-dimensional (3D) flower-like CeO_2_ micro/nanocomposite structure using cerium chloride as a reactant by a simple and economical route based on an ethylene glycol-mediated process [12]. Li et al. synthesized mesoporous Ce(OH)CO_3_ microspheres with flower-like 3D hierarchical structures via different hydrothermal systems, including glucose/acylic acid, fructose/acrylic acid, glucose/propanoic acid, and glucose/n-butylamine systems. Calcination of the Ce(OH)CO_3_ microspheres yielded mesoporous CeO_2_ microspheres with the same flower-like morphology as that of Ce(OH)CO_3_ microspheres [13]. Ouyang et al. reported a facile electrochemical method to prepare hierarchical porous CeO_2_ nanospheres and applied them as highly efficient absorbents to remove organic dyes [14]. However, 3D hierarchical structured CeO_2_ is commonly synthesized with relatively miscellaneous process, which limited the extensive usage of the prepared ceria. In this paper, we report a facile one-pot hydrothermal route to synthesize 3D hierarchical structured CeO_2_. The present hydrothermal route is low cost and can be easily scaled-up. The fabricated 3D hierarchical structured CeO_2_ could be used as a catalyst for CO oxidation and a support for noble metal catalysts.

## 2. Materials and Methods

### 2.1. Preparation of Hierarchical Structured CeO_2_

Cerium (III) chloride heptahydrate (CeCl_3_·7H_2_O), polyvinyl pyrrolidone (PVP), and ethanol were purchased from Beijing Yili Chemical Reagent Co. Ltd. (Beijing, China). All materials were used without any further purification. In a typical synthetic procedure of the hierarchical structured CeO_2_, 0.5 mmol CeCl_3_·7H_2_O was dissolved in 30 mL deionized water, and then 1 mmol PVP and 20 mL deionized water were added to the solution. After 15 min of magnetic stirring, the homogenous solution was transferred into the Teflon vessel of a hydrothermal bomb, which was then placed in an oven and maintained at 270 °C for 24 h. Then, the solution was cooled to room temperature, and the products were separated by centrifugation and washed with absolute ethanol and distilled water.

### 2.2. Characterization Techniques

The crystal phases of the products were characterized by X-ray diffraction (XRD) using Philips X’pert PRO analyzer (Philips, Amsterdam, The Netherlands) equipped with a Cu K_α_ radiation source (λ = 0.154187 nm) and operated at an X-ray tube (Philips, Amsterdam, The Netherlands) voltage and current of 40 KV and 30 mA, respectively. The morphology of the products was examined by scanning electron microscopy (SEM) using a JEOL JSM 67OOF system (JEOL, Tokyo, Japan) and transmission electron microscopy (TEM) using a JEM-2100 system (JEOL, Tokyo, Japan) operated at 200 kV. Surface composition was determined by X-ray photoelectron spectroscopy (XPS) using an ESCALab220i-XL electron spectrometer (VG Scientific, Waltham, MA, USA) with monochromatic Al K_α_ radiation. Nitrogen adsorption-desorption isotherms were analyzed using an automatic adsorption system (Autosorb-1, Quantachrome, Boynton Beach, FL, USA) at the temperature of liquid nitrogen.

## 3. Results

### 3.1. 3D Hierarchical Structured CeO_2_ Prepared via Hydrothermal Method

**Figure 1:**
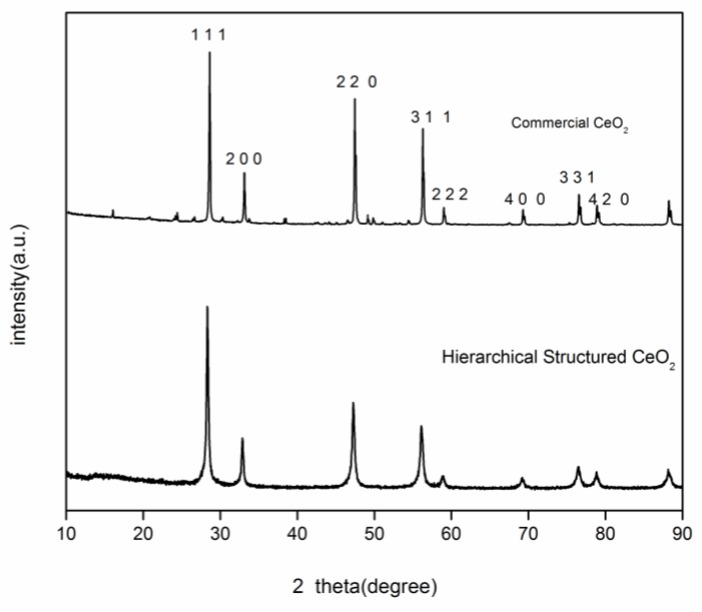
XRD pattern of as-prepared 3D hierarchical structured CeO_2_.

The powder XRD pattern of the as-prepared sample is shown in Figure 1. As can be seen, the as-prepared sample can be indexed to the cubic phase of CeO_2_ (JCPDS No. 34-0394). The average crystallite size calculated by the Scherrer equation is 26.8 nm.

**Figure 2:**
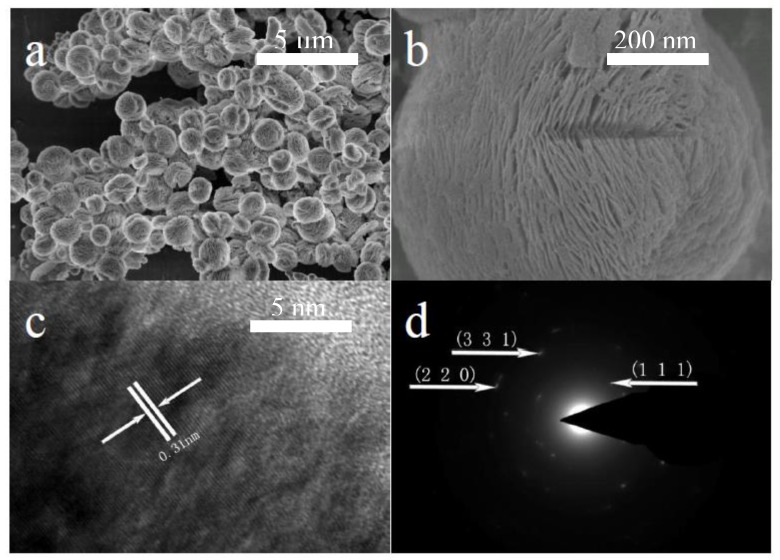
(**a**,**b**) SEM images, (**c**) HRTEM image, and (**d**) SAED pattern of the 3D hierarchical structured CeO_2_.

The SEM images of the as-synthesized CeO_2_ particles are shown in Figure 2. It can be seen from Figure 2a that the as-synthesized CeO_2_ microspheres have diameters of about 1 μm. These CeO_2_ microspheres consist of many nanosheets with thicknesses of about 15 nm. The mesopores with about 20-nm pore sizes are spread over the nanosheets. The lattice fringes in the high-resolution TEM (HRTEM) image (Figure 2c) show a spacing of 0.31 nm, corresponding to the (1 1 1) plane of cubic CeO_2_. The selected area electron diffraction (SAED) pattern (Figure 2d) indicates that the microspheres are composed of low-crystalline CeO_2_ nanocrystals.

**Figure 3:**
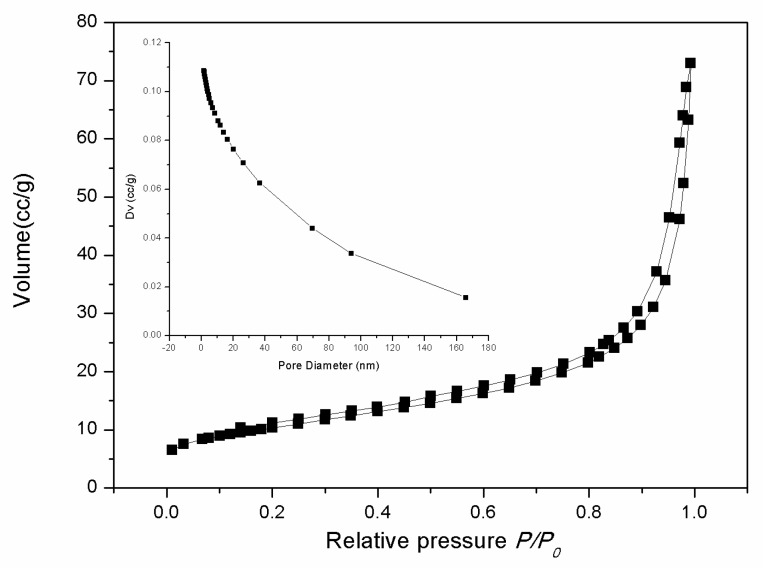
Nitrogen adsorption-desorption isotherm of 3D hierarchical structured CeO_2_. The inset shows the pore size distribution curve obtained from the desorption data.

The nitrogen adsorption and desorption isotherms of the as-prepared samples and the corresponding pore size distribution curve calculated by the Barret-Joyner-Halenda (BJH) method are shown in Figure 3. The nitrogen adsorption and desorption isotherms exhibit a slim hysteresis loop at a relative pressure of >0.2, which is the type-II curve. The calculated Brunauer-Emmett-Teller (BET) surface area of the as-synthesized CeO_2_ is about 36.8 m^2^g^–1^. The average pore size calculated by the BJH method is 14.9 nm.

### 3.2. Effects of Synthesis Conditions on the Formation of 3D Hierarchical Structured CeO_2_ and the Possible Formation Mechanism

**Figure 4:**
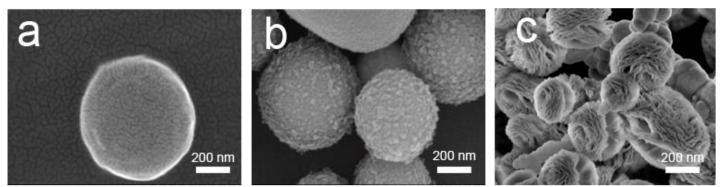
SEM images of CeO_2_ samples prepared at 270 °C for different reaction times: (**a**) 6 h; (**b**) 12 h; and (**c**) 24 h.

**Figure 5:**
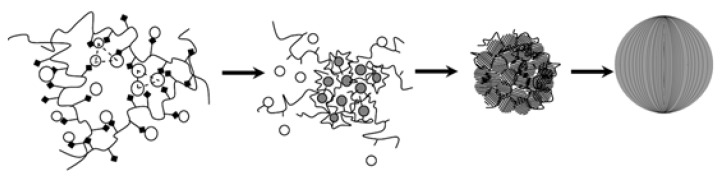
Schematic illustrating the formation of 3D hierarchical structured CeO_2_.

To investigate the evolution of flower-like CeO_2_ particles, the samples obtained after different reaction times were characterized by SEM (Figure 4). The reaction temperature and the dosages of CeCl_3_·7H_2_O and PVP were kept constant (270 °C, 0.01 M, and 0.02 M, respectively). As we can see in Figure 4a, spherical particles were obtained in the early stage. After 12 h of hydrothermal treatment, the sample (Figure 4b) evolved into spheres on which many scrappy grains grew. We speculate that PVP at the surface of the spheres decomposed gradually at such a high temperature and pressure, and simultaneously, tiny nanoparticles on the surface of the spheres began to grow into nanosheets. As seen in Figure 4c, all spheres have transformed into flower-like CeO_2_ particles. Based on these observations, the possible formation mechanism of the 3D hierarchical structured CeO_2_ can be speculated. The schematic mechanism for the 3D hierarchical structured CeO_2_ obtained during different hydrothermal stages is illustrated in Figure 5. At an early stage, Ce^3+^ ions were oxidized gradually by O_2_ present in the aqueous solution to form small CeO_2_ nanocrystals. Then, the small CeO_2_ nanocrystals interacted with PVP and self-assembled as building blocks into spherical particles. As the temperature of the hydrothermal system increased, the PVP at the surface of the spherical particles began to decompose and small nanoparticles began to grow into nanosheets via Ostwald ripening. Due to Ostwald ripening, more were nanosheets formed, and after 24 h of hydrothermal treatment, the PVP completely decomposed and 3D hierarchical structured CeO_2_ particles were formed.

### 3.3. Catalytic Performance of 3D Hierarchical Structured CeO_2_ for CO Combustion

**Figure 6:**
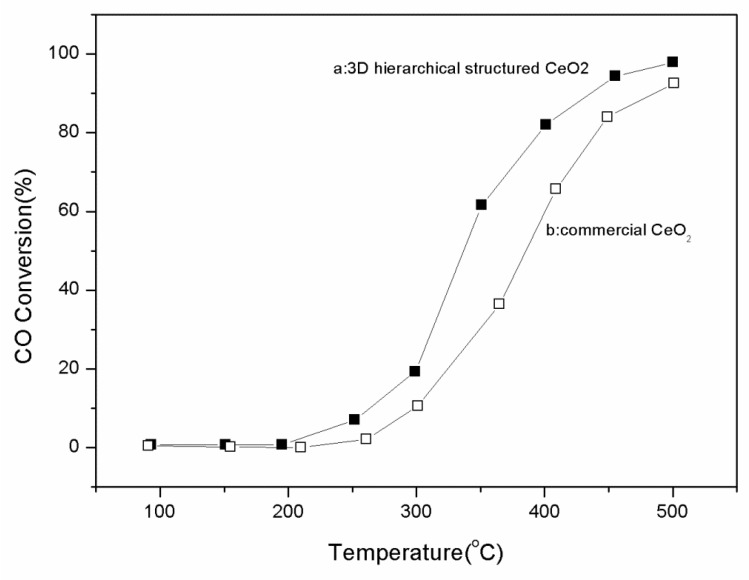
CO conversion rate in the presence of (**a**) as-prepared 3D hierarchical structured CeO_2_, and (**b**) commercial CeO_2_.

Catalytic application is an important direction for CeO_2_ researches because the oxygen storage capacity of CeO_2_ is associated with its ability to undergo a facile conversion between Ce(III) and Ce(IV). Therefore, the catalytic activity of the as-prepared 3D hierarchical structured CeO_2_ was tested by CO oxidation. As shown in Figure 6, the 3D hierarchical structured CeO_2_ exhibits better activity toward CO oxidation than commercial CeO_2_ (purchased from Beijing Yili Chemical Reagent Co. Ltd., Beijing, China) does. The 50% conversion temperature of the 3D hierarchical structured CeO_2_ is about 320 °C, while that of the commercial CeO_2_ is higher than 380 °C.

**Figure 7:**
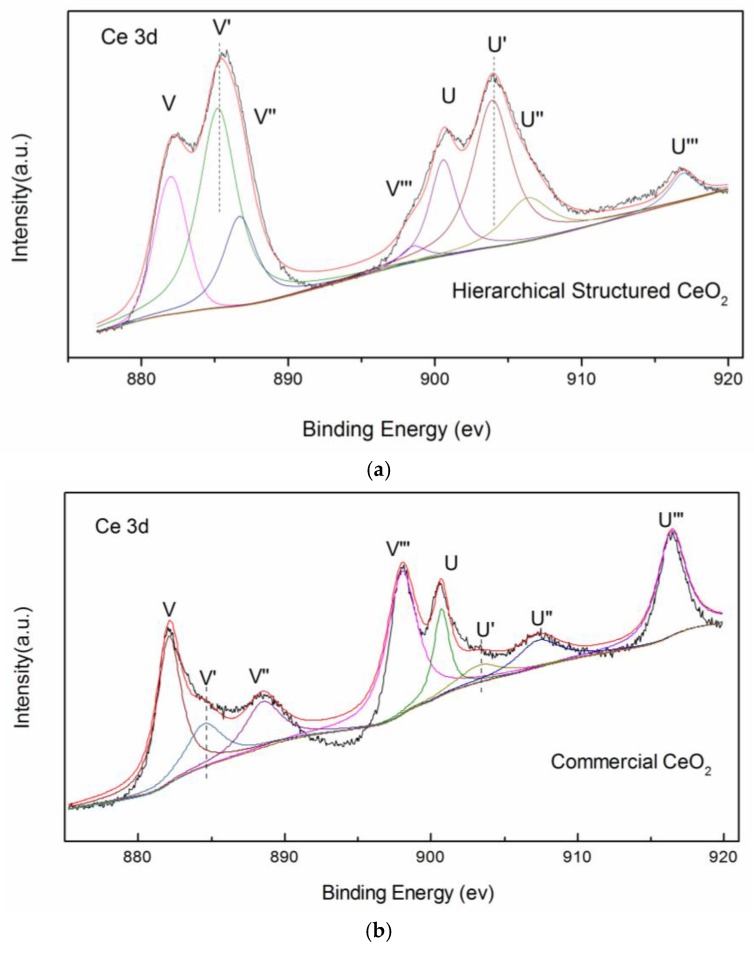
X-ray photoelectron spectra of Ce 3D regions of 3D (**a**) hierarchical structured CeO_2_ and (**b**) commercial CeO_2_.

The sample was further characterized by XPS and the Ce 3d electron core level spectra are shown in Figure 7. The four main 3d_5/2_ features at 882.7, 885.2, 888.5, and 898.3 eV correspond to V, V’, V’’, and V’’’ components, respectively. The 3d_3/2_ features at 901.3, 903.4, 907.3, and 916.9 eV correspond to U, U’, U’’, and U’’’ components [15], respectively. The signals V’ and U’ are characteristic of Ce(III) [16]. According to the ratio of the area for Ce^3+^ peaks to the whole peak area in Ce 3d region, the amount of Ce^3+^ of 3D hierarchical structured CeO_2_ is 51.8%. The amount of Ce^3+^ of commercial CeO_2_ is 13.2%. The 3D hierarchical structured CeO_2_ has a much higher Ce^3+^ concentration, which implies a much higher concentration of oxygen defects compared with commercial CeO_2_. A large amount of oxygen defects enhances the conversion between Ce(III) and Ce(IV), thereby supplying more reactive oxygen. Thus, the special structure of 3D hierarchical structured CeO_2_ provides more active sites for CO oxidation.

## 4. Conclusions

In summary, a simple and economical hydrothermal route was presented to synthesize 3D hierarchical structured CeO_2_ using CeCl_3_·7H_2_O and PVP. The 3D hierarchical structured CeO_2_ has a beautiful flower-like structure, which consists of many nanosheets. A two-stage growth process was identified for the formation of 3D hierarchical structured CeO_2_, and Ostwald ripening was found to play an important role in the transformation of the nanoparticles into nanosheets. The 3D hierarchical structured CeO_2_ exhibited a higher catalytic activity toward CO oxidation compared with commercial CeO_2_.
